# Diphenhydramine-induced delirium on top of HIV-associated neurocognitive disorder

**DOI:** 10.1192/j.eurpsy.2023.860

**Published:** 2023-07-19

**Authors:** R. T. E. Sollano

**Affiliations:** Psychiatry, The Medical City - Ortigas, Pasig City, Philippines

## Abstract

**Introduction:**

HIV-associated neurocognitive disorder has been less frequent in recent years due to the availability of anti-retrovials. However, in the Philippines, persons with HIV are diagnosed late resulting to cases of HIV-associated neurocognitive disorder. With higher incidence of depression and anxiety in this population, difficulty sleeping becomes a prominent symptom and diphenhydramine is a common non-prescription nighttime sleep aid being given.

**Objectives:**

To present a case of diphenhydramine-induced delirium after a patient with HIV-associated neurocognitive disorder.

**Methods:**

This a case report.

**Results:**

Mr. JR., a 38-year-old person living with HIV and no past psychiatric history, presents with acute onset altered mental status, suicidal attempt, and jerking movements of the neck and extremities. He has been having bouts of diarrhea, fatigue, weight loss, and forgetfulness for a year before he was diagnosed with HIV-AIDS with CD4 count of 59 cells/mm3. At this time, he already had blurring of vision, poor sleep, weakness, poor concentration, and increasing severity of forgetfulness. He also started to have depressed mood and anhedonia but no suicidal ideations. He was eventually started on antiretroviral (ARVs) which are lamivudine, tenofovir, dolutegravir and antibiotics targeting opportunistic bacteria – Isoniazid, Moxifloxacin and Clindamycin. A few days after, he started to have jerking movements of the neck and extremities contributing further to poor sleep. Upon consult with a local clinic to address his sleep, he was prescribed with Diphenhydramine and after taking 50mg dose that evening, he started to have disorientation, paranoia, command auditory hallucinations resulting to a suicidal attempt, on top of the jerking movements, which prompted consult to the emergency room and subsequent admission. Initially assessed as central nervous system infection and focal seizure, CSF fluids studies and EEG were done showing normal findings. Started on Sodium Valproate + Valproic acid 500mg IV twice daily and Olanzapine 2.5mg twice a day, on top of his previously mentioned ARVs and antibiotics, the disorientation, auditory hallucinations, and myoclonic jerks mood resolved after five days. Five months on ARVs, he has no recurrence of myoclonic jerks, disorientation or psychosis, with memory and concentration improved, euthymic mood, and was able to resume work as an engineer.

**Conclusions:**

Diphenhydramine is a common nighttime sleep aid. Due to its anticholinergic effect, cases of delirium were reported for doses 300mg to 1,000mg per day. For Mr. JR, the mere 50mg dose of diphenhydramine caused disorientation and psychosis as his co-occurring HIV-associated neurocognitive disorder made his brain “delirium-ready”. Diphenhydramine is a relatively safe drug however not getting a thorough medical history may inadvertently cause harm to patients who are medically ill and frail.

**Disclosure of Interest:**

None Declared

